# 1416. Medicare Spending on Urinary Tract Infections: A Retrospective Database Analysis

**DOI:** 10.1093/ofid/ofab466.1608

**Published:** 2021-12-04

**Authors:** Kate Sulham, Eric Hammelman

**Affiliations:** 1 Spero Therapeutics, Cambridge, MA; 2 Health Management Associates, Chicago, Illinois

## Abstract

**Background:**

Medical visits for UTIs represent 1%-6% of all healthcare visits (~7 million visits) and are estimated to cost the United States (US) healthcare system at least &1.6 billion annually. UTIs are associated with significant morbidity; particularly among the elderly, where UTIs are most prevalent. Little is known about the specific costs to Medicare of UTI; here, we seek to examine overall Medicare spending on UTI.

**Methods:**

We conducted a retrospective multicenter cohort study of the Medicare fee-for-service (FFS) data. Patients were included for analysis if the following criteria were met: (1) enrolled in Medicare FFS from January 1, 2016 through December 31, 2019, (2) not enrolled in Medicare Advantage during that time period, (3) did not have any UTI diagnoses in 2016, and (4) enrolled in Medicare Part D. Individuals were categorized as having uncomplicated UTI (uUTI), complicated UTI (cUTI), or those who first had a uUTI that progressed to a cUTI (uUTI to cUTI). Medicare spending in the 12 months post-diagnosis was calculated, and patients were stratified by home- or institutionally-based (eg, nursing home, long-term care facility, etc.).

**Results:**

2,330,123 patients were included for analysis; 92% were home-based, 8% were institutionally-based. Mean Charlson Comorbidity Index (CCI) across all patients was 2.16. In the 12 months after initial diagnosis, average Medicare spend was &33,984, &9,941 of which was UTI-related. Annual UTI-related costs were approximated &9,000 for home-based vs. &21,444 for institutionally-based patients. Mean drug spend per patient on antibiotics was &872. Broadly, uUTI patients were least expensive, followed by cUTI patients, with uUTI to cUTI patients being most expensive. Higher costs for were observed for institutionally-based patients, largely due to more frequent acute hospitalizations and more Part A-paid skilled nursing stays.

**Conclusion:**

UTI-related spending represents approximately one-third of total annual Medicare spend for patients diagnosed with a UTI. Given average Medicare spending of approximately &12,000 per person in 2019, UTI is associated with substantially increased per patient cost and represents a significant source of spending for Medicare.

**Disclosures:**

**Kate Sulham, MPH**, **Spero Therapeutics** (Consultant) **Eric Hammelman, MBA**, **AbbVie Pharmaceuticals** (Consultant)**Edwards Lifesciences** (Consultant)**Genentech** (Consultant)**Spero Therapeutics** (Consultant)**Vertex Pharmaceuticals** (Consultant)

